# Supplementary material for: Non-saturating quantum magnetization in Weyl semimetal TaAs
Source: Nat Commun. 2019 Mar 4;10:1028. doi: 10.1038/s41467-019-09012-4 (PMC6399290; doi:10.1038/s41467-019-09012-4)
Supplement: Supplementary file 1 — Supplementary Information [file 41467_2019_9012_MOESM1_ESM.pdf]

Supplementary information for "Non-saturating Quantum Magnetization in Weyl semimetal TaAs"

Zhang *et al.*

[a] Corresponding authors: luhz@sustc.edu.cn; zhangjinglei@hmfl.ac.cn and gwljiashuang@pku.edu.cn

Supplementary Note 1: Material and Method

Single crystals of TaAs. Single-crystalline TaAs was grown by a vapor transport method, while the details of crystal growth can be found in Ref. [1]. The single crystal used in the torque measurement is shown in the inset of Supplementary Fig. 1. X-ray diffraction measurements confirmed that the square surfaces are the crystallographic **c** planes with four-fold rotational axes. The angle $\theta = 0°$ is defined as the direction of the magnetic field which coincides with **c** axis. The temperature-dependent resistivity (Supplementary Fig. 1) was performed in a Quantum Design physical property measurement system (PPMS-9). A standard four-probe method for resistivity measurements was adopted with employing silver paste contacts.

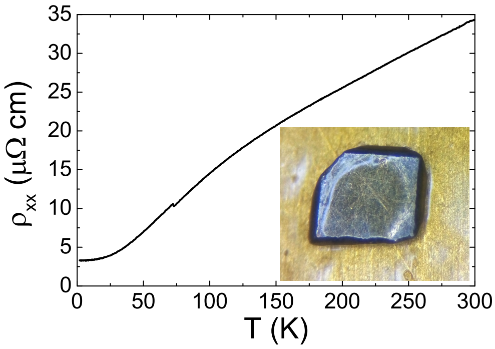

Supplementary Fig. 1. Temperature-dependent resistivity of the sample of TaAs used in magnetic torque. The inset shows its image with a size of 0.5×0.5 mm^2.

Cantilever calibration and background. In order to measure the magnetic torque signal precisely in an intense magnetic field, we adopted a capacitive method in this experiment which is well-known as a sensitive method with small background signal. A simple sketch (Supplementary Fig. 2a) shows the construction of the capacitive cantilever setup. An off-axial magnetic field exerts an external torsional force on the sample, which subsequently causes the deflection of the cantilever. As deflected by the torque, the cantilever has a change of the distance which can be detected by a capacitance bridge.

In order to estimate the value of magnetic torque from the raw capacitance data, we calibrated the cantilever to obtain the spring constant κ. We used a mass-changeable weight to test the cantilever for the avoidance of the angle-dependent background contribution of parasitic capacitance [2, 3]. The setup is shown in Supplementary Fig. 2b, where the whole

probe is horizontally put on ground. In zero magnetic field, the torque equals Mgl where l is the length of cantilever. The torques caused by the weights (Mg) with different masses are counterbalanced by $\kappa\delta\theta$, where $\delta\theta$ is the small angle caused by cantilever deflection. In the case of small-angle deflection approximation [3], the fractional change in capacitance $\delta C/C_0$ can be expressed as

$$\frac{\delta C}{C_0} = \frac{Mgl^2}{\kappa d_0}, \tag{1}$$

where magnetic torque is expressed as τ = -$\kappa\delta\theta$ and d_0 is the zero-torque capacitance gap between the two metal foils.

By varying the mass of weight, different values of $\delta C/C_0$ were obtained and subsequently plotted against Mgl^2/d_0 as shown in Supplementary Fig. 2c. A linear fit to these raw data gives a value of κ to be 8.5×10^{-5} N m/rad.

Quite generally, a material with magnetization **M**, in a magnetic field **B**, will experience a torque $\tau = \mathbf{M} \times \boldsymbol{\mu_0}\mathbf{H} + \boldsymbol{\mu_0}\mathbf{M}\nabla\mathbf{H} \times \mathbf{d}$ where d is the distance from the sample to the torque axis. The first term is due to the anisotropic magnetization and depends on the component of M perpendicular to the applied field direction. The second term $\mu_0\mathbf{M}\nabla\mathbf{H} \times \mathbf{d}$ comes from the isotropic magnetization of the sample and leads to a force on the sample in a magnetic field gradient. In our studies the sample size is smaller than 0.5 mm×0.5 mm×0.2 mm and the field homogeneity is better than 450 ppm/dsv. In such case the magnetic force is negligible and the cantilever gives the pure torque signal $\mathbf{M} \times \boldsymbol{\mu_0}\mathbf{H}$. Our measurements were carried out on a capacitive cantilever in a steady high field facility. Basically, the torque signal is in proportion to the small angle caused by cantilever deflection $\tau = \kappa d\theta$. However we need to point out that such approximation would be invalid if the torque signal is too larger. As shown in Supplementary Fig. 2c, the capacitance increases linearly with torque signal as it reaches to 1.6×10^{-6} N/m at least. Besides the maximum torque signal for our TaAs sample is around 1.2×10^{-6} N/m. It indicates that in our studies the cantilever is in the linear elastic range.

A caveat for the torque measurement in strong magnetic field is the existence of a large, nonlinear background. This kind of measurement error due to improper setup should be seriously considered in piezo-based cantilever setup, especially in a pulsed magnetic field. In contrast, our measurements were carried out on a capacitive cantilever in a steady high field facility. In order to role out the possibility of the background signal, we measured an empty torque in a strong constant field. As shown in Supplementary Fig. 3, the background

is smooth and ignorable compared with the small-angle experimental data of TaAs.

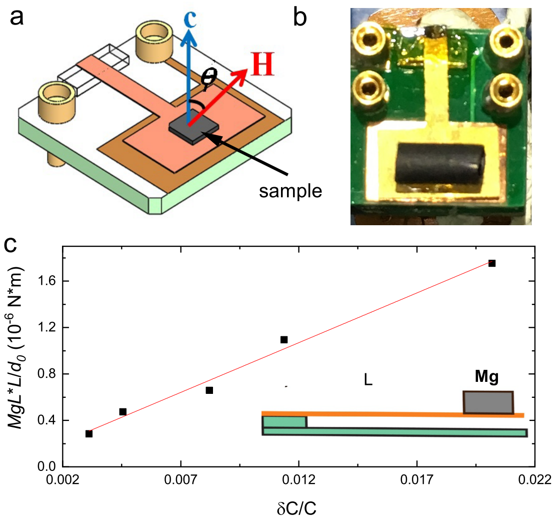

Supplementary Fig. 2. **Setup and calibration for the magnetic torque cantilever.** **a** A sketch of the torque setup. **b** A photo of the capacitive cantilever setup; the black item is the weight for calibration. The setup is horizontally placed out of dewar. **c** The linear fitting for obtaining the spring constant κ.

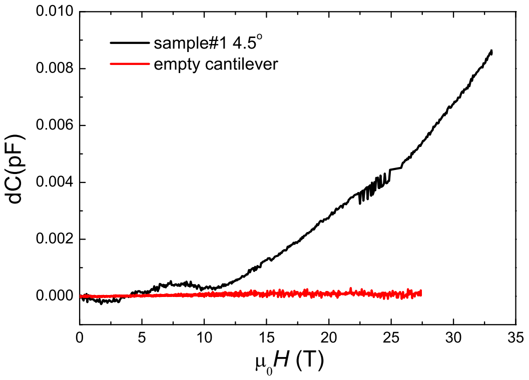

Supplementary Fig. 3. The empty cantilever shows a small and smooth background compared with the raw data when the sample is there.

Supplementary Note 2: Theory of field-dependent magnetization for parabolic and Weyl bands

Field-dependent magnetization for a single parabolic band — a fundamental approach. Traditional theory solves the problem of Landau diamagnetism and Lifshitz-Kosevich (L-K) formula in a small magnetic field. In order to comprehend the magnetization in strong magnetic field, we focus on the case when few of Landau bands are occupied below the Fermi level (FL).

In the thermodynamic potential treatment in statistical mechanics for magnetization (as shown in [4] by Shoenberg), the magnetic moment vector is given by

$$\mathbf{M} = -(\nabla_{\mathbf{B}} \varOmega)_{E_{\mathrm{F}}}, \tag{2}$$

where E_{F} is the FL.

In more practical terms, the components of $\mathbf{M}$ parallel and perpendicular to the magnetic field $\mathbf{B}$ as

$$M_{\parallel} = -(\frac{\partial \varOmega}{\partial B})_{E_{\mathrm{F}}}; M_T = -\frac{1}{B}(\frac{\partial \varOmega}{\partial \theta})_{E_{\mathrm{F}},B}, \tag{3}$$

and the magnetic torque is defined as $\tau = -(\frac{\partial \varOmega}{\partial \theta})_{E_{\mathrm{F}},B}$.

For a Fermi-Dirac (FD) statistical system which has states of energy ϵ_i, the thermodynamics potential is given by

$$\varOmega = -k_B T \sum_{\epsilon_i} \ln(1 + e^{(E_{\mathrm{F}}-\epsilon_i)/k_B T}), \tag{4}$$

with k_B is the Boltzmann constant.

Here we consider a three-dimensional (3D) system case and $T = 0$ K, selecting the z-term as the free momentum. Performing the integral on this direction, we can rewrite the Supplementary Eq. (4) as

$$\varOmega = -\int_{-\infty}^{+\infty} d\mathbf{k} \frac{eBV}{2\pi^2 \hbar} \sum_n (\epsilon_n - E_{\mathrm{F}}), \tag{5}$$

where the degeneracy of the one-dimensional (1D) Landau tube under a magnetic field is defined as $D = g_s \frac{eBV}{2\pi\hbar} \frac{dk_z}{2\pi}$.

Then we first consider a parabolic band with an ellipsoidal Fermi surface (FS), $\epsilon(\mathbf{k}) = \frac{\hbar^2}{2}[\frac{k_x^2+k_y^2}{m_p} + \frac{k_z^2}{m_z}]$. Under an external magnetic field, the Landau Band (LB) spectrum is

$$\epsilon(\mathbf{k}) = (n+\gamma)\hbar\omega_c + \frac{\hbar^2 k_z^2}{2m_z}, \quad n = 0, 1, 2, \cdots \tag{6}$$

Inserting Supplementary Eq. (6) into (5), we can obtain the final version of thermodynamics potential of a parabolic band, where the up-limit of the integral performing on the 1D LL tube is given by the constraint as $\frac{\hbar^2 k_z^2}{2m_z} = E_{\mathrm{F}} - (n+\gamma)\hbar\omega_c$. We find

$$\Omega = -g\frac{eV}{3\pi^2\hbar}B\frac{\sqrt{2m_z}}{\hbar}\sum_n [E_{\mathrm{F}} - (n+\gamma)\hbar\omega_c]^{3/2}, \quad (7)$$

By the first order derivative to the magnetic field, we can obtain the expression of $M_{\|}$

$$M_{\|} = -\frac{\partial\Omega}{\partial B} = g\frac{eV}{3\pi^2\hbar}\frac{\sqrt{2m_z}}{\hbar}\sum_n [E_{\mathrm{F}} - (n+\gamma)\hbar\omega_c]^{1/2}[E_{\mathrm{F}} - \frac{5}{2}(n+\gamma)\hbar\omega_c], \quad (8)$$

If we hold the FL(E_{F}) as a constant, we obtain the result as shown in Supplementary Fig. 4a. In the high-field regime, the simulation well reproduces the dHvA oscillations when many LBs are located below the FL. The bottom of N = 1 LB leaves the FL beyond the quantum limit (QL) where only the last LB (N = 0) remains below the FL. In the constrain of a fixed FL, the energy of the last LB will exceed the Fermi energy in a sufficiently strong field. In this situation, the $M_{\|}$ shows broad peak at $\mu_0 H = 70$ T and zero value above 90 T. However this situation is unlikely to occur in real materials no matter they are topological or not. A constraint for the real materials in such extreme magnetic fields is that the last LB will be pinned at the FL due to the conservation of the carrier concentration (N_c). Therefore the FL is inevitably changed in strong field. In order to calculate the magnetization in the QL, we followed the treatment in ref. [5] which constrains N_c as a constant.

When the system enters the QL, all carriers are confined in the last LB, and $N_c = 4eBp_z/h^2$. The total energy can be written as

$$E_{\mathrm{tot}} = \mu N_c B + \frac{N_c^3}{32m}\frac{h^4}{e^2}\frac{1}{B^2}, \quad (9)$$

where $\mu = \frac{e\hbar}{2m}$ and N_c holds constant. Then it is easy to see the magnetization in the QL is

$$M_{\|} = -\mu N_c + \frac{N_c^2}{16m}\frac{h^4}{e^2}\frac{1}{B^3}. \quad (10)$$

This result is shown as the black line beyond the QL in Supplementary Fig. 4a. We can see the magnetization in real material with a trivial parabolic band will follow an B^{-3} dependence beyond QL and saturate at higher fields.

To check the results of magnetic torque, we follow a similar analysis within the small-angle approximation [2]. By differentiating the angle θ, the ω_c in the summand contributes

the main order, and the torque follows

$$\tau = -\frac{\partial\Omega}{\partial\theta} = g\frac{eV\omega_c\theta}{2\pi^2\hbar}B\sqrt{2m_z}\sum_n[E_{\mathrm{F}} - (n+\gamma)\hbar\omega_c]^{1/2}(n+\gamma). \tag{11}$$

The effective magnetization is $M_T(\text{volume}) = \tau/BV$ with the volume V. These two field-dependent quantities are plotted in Supplementary Figs. 4b and 4c, respectively. The number of electron in the LBs from 0th to nth is

$$N_{cn} = V \cdot D_{2D}\int_{-k_z^m}^{+k_z^m}\frac{dk_z}{2\pi}, \tag{12}$$

where $D_{2D} = eBV/\pi\hbar$, and $k_z^m = \frac{\sqrt{2m_z(E_{\mathrm{F}}-(n+\gamma)\hbar\omega_c)}}{\hbar}$. When the system enters the QL, $N_c = N_{c0}$, then by simple calculation, we can find

$$M_T(LLL) = g\frac{e^2\omega_c\theta}{2\pi^2\hbar}\sqrt{2m_z}[E_{\mathrm{F}} - \gamma\hbar\omega_c]^{1/2}\gamma = \frac{e\theta}{2m_p}\gamma N_{c0} \tag{13}$$

which is essentially a constant, so the red lines in the original formula are replaced by violet lines in Supplementary Figs. 4b and 4c for a fixed N_c .

Field-dependent magnetization for Weyl electron gas. Now we consider a simple model of Weyl fermions with anisotropic Fermi velocities. For Weyl semimetals, the low energy excitations always consist of a pair of Weyl fermions with opposite chiralities. The Hamiltonian reads $H_W = \chi H$ with $\chi = \pm 1$ and

$$H = v_a p_x\sigma_x + v_b p_y\sigma_y + v_c p_z\sigma_z, \tag{14}$$

where v_a, v_b, and v_c are the Fermi velocities; $(\sigma_x, \sigma_y, \sigma_z)$ are Pauli matrices. If the magnetic field is applied in x-z plane $\mathbf{B} = (B\sin\theta, 0, B\cos\theta)$, then the vector potential is

$$\mathbf{A} = (-By\cos\theta, 0, By\sin\theta). \tag{15}$$

The energies of the Landau bands of Weyl fermions with chirality χ are given by

$$E_0^W = -\,\chi v_b k_\parallel = \chi E_0, \tag{16}$$

$$E_{n\pm}^W = \pm\,\chi v_b\sqrt{2n\Delta eB + k_\parallel^2} = \chi E_{n-}, \qquad n \geq 1, \tag{17}$$

where

$$k_\parallel = \frac{v_c}{v_b}\cos\alpha k_z + \frac{v_a}{v_b}\sin\alpha k_x, \tag{18}$$

$$\alpha = \tan^{-1}\left(\frac{v_c}{v_a}\tan\theta\right), \tag{19}$$

$$\Delta = \frac{v_a}{v_b}\cos\alpha\cos\theta + \frac{v_c}{v_b}\sin\alpha\sin\theta. \tag{20}$$

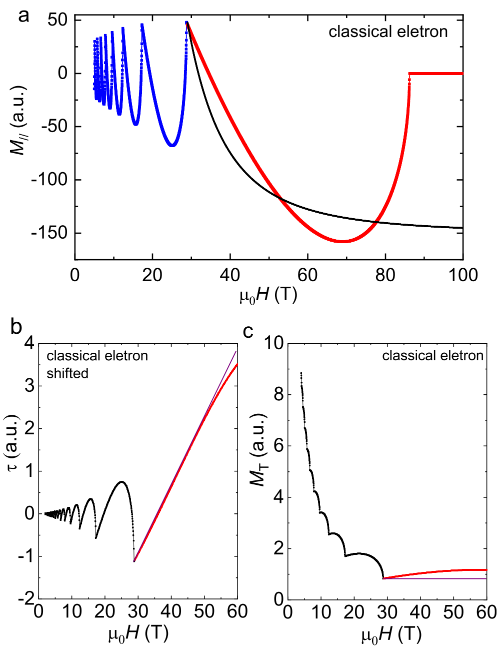

Supplementary Fig. 4. **Magnetization and magnetic torque of free electrons in the QL in theory.** **a** Theoretical high-field $M_{||}$. Red line shows result in pristine formula; black line shows the result under the constraint of fixed N_c. **b** Theoretical high-field τ and **c** converted M_T, red line shows result in pristine formula; violet line shows the result under the constraint of fixed N_c.

Further, it could be found that

$$\frac{\partial \Delta(\theta)}{\partial \theta} = \frac{(v_c^2 - v_a^2)\sin\theta}{v_b\sqrt{v_c^2\tan^2\theta + v_a^2}}, \tag{21}$$

which exists when $v_a \neq v_c$.

The thermodynamic potential is

$$\Omega(B) = -k_B T\int_{-\infty}^{\infty} dE \ln\left[1 + e^{(E_\mathrm{F}-E)/k_B T}\right]\frac{dW(E)}{dE}, \tag{22}$$

where $W(E)$ is the total states and $\frac{dW(E)}{dE}$ is the density of states.

For the positive energy bands, the thermodynamic potential is

$$\Omega_c(B) = \int_0^{\infty} dE \frac{dn_F(E)}{dE}\left[\int_0^E dE' W(E')\right] \tag{23}$$

where $n_F(E)$ is the Fermi distribution function. At zero temperature, it can be obtained as

$$\Omega_c(B) = -\frac{\Delta e B E_\mathrm{F}^2}{8\pi^2 v_b}\left[\frac{E_\mathrm{F}^2}{3v_b^2\Delta e B} - 1\right] - \frac{v_b}{2\pi^3}(\Delta e B)^2\sum_{s=1} s^{-3/2}\int_0^{\bar{M}} dM f_\mathrm{os}(s, M), \tag{24}$$

with $\bar{M} = E_{\mathrm{F}}/(v_b\sqrt{2\Delta eB})$ and $f_{\mathrm{os}}(s, M) = \sin(2\pi sM^2)\mathrm{Fc}(2\sqrt{s}M) - \cos(2\pi sM^2)\mathrm{Fs}(2\sqrt{s}M)$.

For the negative energy bands, we assume the lowest energy of valence band is $-\Lambda$, i.e. $E_{n-} \geq -\Lambda$, and the thermodynamic potential $\Omega_v(B)$ is

$$\Omega_v(B) = \frac{\Delta eB}{\pi} \sum_{n,k_\parallel} (E_{n-} - E_{\mathrm{F}})\Theta(\Lambda + E_{n-}), \tag{25}$$

with $\Theta(x)$ being the step function. The major contributions can be calculated as [6]

$$\Omega_v(B) \sim \frac{\Lambda^4}{24\pi^2 v_b^3} + \frac{v_b(\Delta eB)^2}{48\pi^2}\left(\ln\frac{2\Lambda}{\sqrt{2v_b^2\Delta eB}} + \mathcal{A}\right), \tag{26}$$

with the constant

$$\mathcal{A} = \frac{1}{2}\left(\ln 2\pi + C_{EM}\right) + \frac{3}{\pi^3}\sum_{s=1}^{\infty}\frac{\ln s}{s^2} \approx 1.49. \tag{27}$$

Here $C_{EM} \sim 0.5772$ is the Euler-Mascheroni constant.

For the case of fixed Fermi energy, from the definitions of the vector magnetic moments Supplementary Eq. (3) the contributions of positive bands $M_{c,\parallel}$, $M_{c,T}$ and contributions of negative bands $M_{v,\parallel}$, $M_{v,T}$ are expressed as

$$M_{c,\parallel} = -\frac{\Delta E_{\mathrm{F}}^2}{8\pi^2 v_b \ell_B^2 B} - \frac{\sqrt{2\Delta}\Delta E_{\mathrm{F}}}{8\pi^3\ell_B^3 B} f_{\mathrm{os}}(1, \bar{M}) + \frac{v_b\Delta^2}{\pi^3\ell_B^4 B}\int_0^{\bar{M}} dM f_{\mathrm{os}}(1, M), \tag{28}$$

$$M_{c,T} = \left[-\frac{E_{\mathrm{F}}^2}{8\pi^2 v_b \ell_B^2 B} - \frac{\sqrt{2\Delta}E_{\mathrm{F}}}{8\pi^3\ell_B^3 B} f_{\mathrm{os}}(1, \bar{M}) + \frac{v_b\Delta}{\pi^3\ell_B^4 B}\int_0^{\bar{M}} dM f_{\mathrm{os}}(1, M)\right]\frac{\partial\Delta(\theta)}{\partial\theta}, \tag{29}$$

$$M_{v,\parallel} = -\frac{v_b\Delta^2}{24\pi^2\ell_B^4 B}\left[\ln\left(\frac{2\Lambda}{v_b\sqrt{2\Delta eB}}\right) + \mathcal{A} - \frac{1}{4}\right], \tag{30}$$

$$M_{v,T} = -\frac{v_b\Delta}{24\pi^2\ell_B^4 B}\left[\ln\left(\frac{2\Lambda}{v_b\sqrt{2\Delta eB}}\right) + \mathcal{A} - \frac{1}{4}\right]\frac{\partial\Delta(\theta)}{\partial\theta}, \tag{31}$$

with magnetic length $\ell_B = \sqrt{\hbar/eB}$.

While for the case when the electron density N_c is fixed, the Fermi energy E_{F} may depend on both the magnitude and the direction of the magnetic field. The total electron density reads

$$N_c = \frac{\Delta}{2\pi\ell_B^2}\left[\sum_{n=1,k_\parallel} 2f(E_{n+}) + \sum_{k_\parallel} f(E_0)\right], \tag{32}$$

with $f(x) = 1/\{\exp[(x - E_{\mathrm{F}})/k_B T] + 1\}$ being the Fermi distribution function.

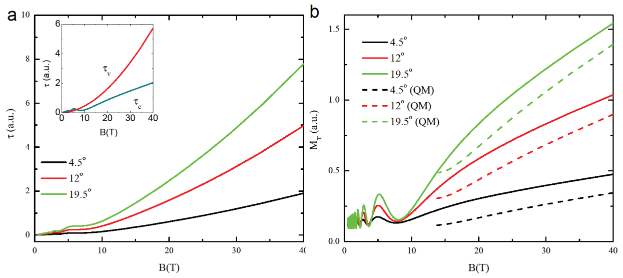

Supplementary Fig. 5. Calculated τ **a** and M_T **b** as functions of magnetic field in different directions. $M_{\|}$ in QL for fixed N_c is shown with dash line. Parameters: $E_{\mathrm{F}} = 0.07$ eV, $v_b = 0.5$ eVnm, $\lambda = 0.4$, and $\Lambda = 0.4$ eV.

It is difficult to derive the expressions of magnetic moments in the general case. Here we give them in the QL, i.e., only the lowest band is occupied. In this situation, the Fermi energy

$$E_{\mathrm{F}} = 2\pi^2 v_b \ell_B^2 N_c/\Delta, \tag{33}$$

is inversely proportional to the magnitude of magnetic field but irrelevant to its direction. In the QL $\bar{M} = E_{\mathrm{F}}\ell_B/(v_b\sqrt{2\Delta}) \ll 1$, we have

$$M_{c,\|} = \frac{\pi^2 v_b N_c^2 \ell_B^2}{2\Delta B} - \frac{8\pi^6 v_b N_c^4 \ell_B^8}{\Delta^4 B}, \tag{34}$$

$$M_{v,\|} = -\frac{v_b \Delta^2}{24\pi^2 \ell_B^4 B}\left[\ln\left(\frac{2\Lambda\ell_B}{v_b\sqrt{2\Delta}}\right) + \mathcal{A} - \frac{1}{4}\right]. \tag{35}$$

It is reasonable that Λ is very large compared with the Fermi energy. Therefore, the total $M_{\|}$ in the QL

$$M_{\|} \propto -B\ln[2\Lambda\ell_B/(v_b\sqrt{2\Delta})], \tag{36}$$

which actually is correct for both constrains of the fixed FL and N_c.

For perpendicular component in the QL,

$$M_{c,T} = \left(-\frac{8\pi^6 v_b \ell_B^8 N_c^4}{\Delta^5 B} - \frac{\pi^4 v_b^2 \ell_B^4 N_c^3}{\Delta^3 B} + \frac{\pi^2 v_b \ell_B^2 N_c^2}{\Delta^2 B}\right)\frac{\partial\Delta(\theta)}{\partial\theta} \propto B^{-2}\frac{\partial\Delta(\theta)}{\partial\theta}, \tag{37}$$

$$M_{v,T} \sim -\frac{v_b\Delta}{24\pi^2\ell_B^4 B}\ln\left(\frac{2\Lambda\ell_B}{v_b\sqrt{2\Delta}}\right)\frac{\partial\Delta(\theta)}{\partial\theta} \propto -B\ln\left(\frac{2\Lambda\ell_B}{v_b\sqrt{2\Delta}}\right)\frac{\partial\Delta(\theta)}{\partial\theta}. \tag{38}$$

Therefore, the total M_T in the QL

$$M_T \propto -B \ln\left(\frac{2\Lambda\ell_B}{v_b\sqrt{2\Delta}}\right)\frac{\partial\Delta(\theta)}{\partial\theta}, \tag{39}$$

which is also correct for the fixed Fermi energy case.

In a numerical simulation, we set $v_c = \lambda v_{a,b}$, then

$$\Delta = \cos\alpha\cos\theta + \lambda\sin\alpha\sin\theta, \tag{40}$$

$$\frac{\partial\Delta(\theta)}{\partial\theta} = \frac{(\lambda^2-1)\sin\theta}{\sqrt{\lambda^2\tan^2\theta+1}}. \tag{41}$$

with $\alpha = \tan^{-1}(\lambda\tan\theta)$.

The calculated $M_\parallel$, M_T, and τ for the fixed FL and N_c in the QL are shown in Supplementary Fig. 5. It is palpable that τ and M_T are enhanced when the system is beyond the QL. This enhancement is due to the contribution of $M_{v,T}$ in the term of $-B\ln[2\Lambda\ell_B/(v_b\sqrt{2\Delta})]$, which could be seen in the inset of Supplementary Fig. 5a.

Field-dependent magnetization for a gapped or gapless trivial electron gas. Now we consider a trivial electron gas with a gap Δ_g

$$H = \begin{bmatrix} H_e & 0 \\ 0 & H_h \end{bmatrix} = \begin{bmatrix} \frac{\hbar^2k_x^2}{2m_{ex}} + \frac{\hbar^2k_y^2}{2m_{ey}} + \frac{\hbar^2k_z^2}{2m_{ez}} + \frac{\Delta_g}{2} & 0 \\ 0 & -\frac{\hbar^2k_x^2}{2m_{hx}} - \frac{\hbar^2k_y^2}{2m_{hy}} - \frac{\hbar^2k_z^2}{2m_{hz}} - \frac{\Delta_g}{2}. \end{bmatrix} \tag{42}$$

The magnetic field also is applied in the x-z plane $\mathbf{B} = (B\sin\theta, 0, B\cos\theta)$, then the vector potential is

$$\mathbf{A} = (-By\cos\theta, 0, By\sin\theta). \tag{43}$$

In the presence of the magnetic field, the Landau bands of positive energies are

$$E_\nu^e = \hbar\omega_e\left(\nu+\frac{1}{2}\right) + \frac{\hbar^2}{2m_{ey}}k_{ez}^2 + \frac{\Delta_g}{2}, \qquad \nu = 0,1,2,\cdots, \tag{44}$$

with $\omega_e = e\Delta_e B/m_{ey}$ and

$$\Delta_e = \sqrt{\frac{m_{ey}}{m_{ex}}\cos^2\theta + \frac{m_{ey}}{m_{ez}}\sin^2\theta}, \tag{45}$$

$$k_{ez} = \frac{1}{\Delta_e}\frac{m_{ey}}{\sqrt{m_{ex}m_{ez}}}\left(k_x\sin\theta + k_z\cos\theta\right). \tag{46}$$

Similarly, the Landau bands of negative energies are

$$E^h_\nu = -\hbar\omega_h\left(\nu+\frac{1}{2}\right) - \frac{\hbar^2}{2m_{hy}}k^2_{hz} - \frac{\Delta_g}{2}, \quad \nu = 0,1,2,\cdots, \tag{47}$$

with $\omega_h = e\Delta_h B/m_{hy}$ and

$$\Delta_h = \sqrt{\frac{m_{hy}}{m_{hx}}\cos^2\theta + \frac{m_{hy}}{m_{hz}}\sin^2\theta}, \tag{48}$$

$$k_{hz} = \frac{1}{\Delta_h}\frac{m_{hy}}{\sqrt{m_{hx}m_{hz}}}\left(k_x\sin\theta + k_z\cos\theta\right). \tag{49}$$

For a fixed electron density in the quantum limit, the magnetization of the contribution from the conduction bands is given by

$$\begin{aligned} M_{c,\parallel} =& -\frac{4\sqrt{2}\pi^2\hbar^2N_c^2}{3m_{ey}B}\ell_e\bar{M}^3 + \frac{5\sqrt{2}\hbar^2}{8\pi^3m_{ey}\ell_e^5B}\sum_{s=1}s^{-3/2}\int_0^{\bar{M}}dMMf_{\rm os}(s,M) \\ & - \frac{\sqrt{2}\hbar^2}{8\pi^3m_{ey}\ell_e^5B}\left(\bar{M}^2+\frac{1}{2}\right)\sum_{s=1}s^{-3/2}f_{\rm os}(s,\bar{M}) - \frac{\sqrt{2}\hbar^2}{16\pi^3m_{ey}\ell_e^3B}\left(8\pi^4N_c^2\ell_e^4 - \frac{1}{\ell_e^2}\right) \\ & \times\sum_{s=1}s^{-3/2}f_{\rm os}(s,\bar{M}), \end{aligned} \tag{50}$$

and

$$M_{c,T} = \frac{1}{\Delta_e}\frac{\partial\Delta_e}{\partial\theta}M_{c,\parallel}. \tag{51}$$

Here $\ell_e^2 = \hbar/e\Delta_e B$,

$$\bar{M} = \sqrt{\frac{1}{\hbar\omega_e}\left(E_{\rm F} - \frac{\Delta_g}{2} - \frac{1}{2}\hbar\omega_e\right)}, \tag{52}$$

$$E_{\rm F} = \frac{\hbar^2}{2m_{ey}}\left(4\pi^4N_c^2\ell_e^4 + \frac{1}{\ell_e^2}\right) + \frac{\Delta_g}{2}. \tag{53}$$

For the contribution from the valence bands,

$$M_{v,\parallel} = \frac{\sqrt{2}\hbar^2}{4\pi^2m_{hy}\ell_h^5}\frac{1}{B}\left[\frac{2}{3}\bar{n}^{3/2} - \frac{\sqrt{\bar{n}}}{3} + \frac{1}{24\sqrt{\bar{n}}}\right] + N_v\frac{\partial E_{\rm F}}{\partial B}, \tag{54}$$

$$= \frac{\sqrt{2}\hbar^2}{4\pi^2m_{hy}\ell_h^5}\frac{1}{B}\left[\frac{2}{3}\bar{n}^{3/2} - \frac{\sqrt{\bar{n}}}{3} + \frac{1}{24\sqrt{\bar{n}}}\right] - N_v\frac{\hbar^2}{2m_{ey}B}\left(8\pi^4N_c^2\ell_e^4 - \frac{1}{\ell_e^2}\right). \tag{55}$$

And still

$$M_{v,T} = \frac{1}{\Delta_h}\frac{\partial\Delta_h}{\partial\theta}M_{v,\parallel}. \tag{56}$$

Here $\ell_h^2 = \hbar/e\Delta_h B$,

$$\bar{n} =\frac{1}{\hbar\omega_h}\left(\Lambda - \frac{\Delta_g}{2}\right) - \frac{1}{2}. \tag{57}$$

For the first term, because

$$\bar{n} =\frac{1}{\hbar\omega_h}\left(\Lambda - \frac{\Delta_g}{2}\right) - \frac{1}{2}, \tag{58}$$

$$\sim\frac{1}{\hbar\omega_h}\left(\Lambda - \frac{\Delta_g}{2}\right) \propto \frac{1}{B}, \tag{59}$$

we have

$$\frac{\sqrt{2}\hbar^2}{4\pi^2 m_{hy}\ell_h^5}\frac{1}{B}\left[\frac{2}{3}\bar{n}^{3/2} - \frac{\sqrt{\bar{n}}}{3} + \frac{1}{24\sqrt{\bar{n}}}\right] \sim\frac{\sqrt{2}\hbar^2}{4\pi^2 m_{hy}\ell_h^5}\frac{1}{B}\frac{2}{3}\bar{n}^{3/2}, \tag{60}$$

$$=\frac{\sqrt{2}\hbar^2}{6\pi^2 m_{hy}\ell_h^5}\frac{1}{B}\bar{n}^{3/2} = \text{constant}. \tag{61}$$

For the second term at very large magnetic fields $8\pi^4 N_c^2 \ell_e^4 \ll \ell_e^{-2}$, then

$$-N_v\frac{\hbar^2}{2m_{ey}B}\left(8\pi^4 N_c^2 \ell_e^4 - \frac{1}{\ell_e^2}\right) \sim N_v\frac{\hbar^2}{2m_{ey}B}\frac{1}{\ell_e^2} = \text{constant}. \tag{62}$$

Therefore, $M_{v,\|} \to$ constant at large magnetic fields. This saturation is also correct for the case of gapless trivial bands as sketched in Fig. 1 in the main text. The total $M_\|$ for this gapped or gapless trivial electron gas is shown in Supplementary Fig. 6.

Field-dependent magnetization for a Dirac model with gap. The Hamiltonian for the gapped Weyl semimetals is

$$H_G = (v_a p_x \sigma_x + v_b p_y \sigma_y + v_c p_z \sigma_z)\tau_x + m\tau_z. \tag{63}$$

The magnetic field is applied in the x-z plane $\mathbf{B} = (B\sin\theta, 0, B\cos\theta)$, then the vector potential is

$$\mathbf{A} = (-By\cos\theta, 0, By\sin\theta). \tag{64}$$

In the presence of the magnetic field, the Hamiltonian becomes

$$H_G = [v_a(k_x - eBy\cos\theta)\sigma_x + v_b p_y \sigma_y + v_c(k_z + eBy\sin\theta)\sigma_z]\tau_x + m\tau_z. \tag{65}$$

with $p_y = -i\partial_y$. The Landau bands are given by

$$E_0^s(k_\|) =s\sqrt{m^2 + v_b^2 k_\|^2}, \tag{66}$$

$$E_\nu^{ss'}(k_\|) =ss'\sqrt{2\nu v_b^2/\ell_{B'}^2 + m^2 + v_b^2 k_\|^2}, \qquad \nu \geq 1 \tag{67}$$

with $s = s' = \pm 1$. Here $\ell_{B'}^2 = \hbar/(e\Delta B)$ and

$$k_\parallel = \frac{v_a}{v_b}\sin\alpha k_x + \frac{v_c}{v_b}\cos\alpha k_z, \tag{68}$$

$$\alpha = \tan^{-1}\left(\frac{v_c}{v_a}\tan\theta\right), \tag{69}$$

$$\Delta = \frac{v_a}{v_b}\cos\alpha\cos\theta + \frac{v_c}{v_b}\sin\alpha\sin\theta. \tag{70}$$

For a fixed electron density N_c in the quantum limit, the magnetization of the conduction bands $M_{c,\parallel}$ is given by

$$\begin{aligned} M_{c,\parallel} =& \frac{v_b}{\pi^2\ell_{B'}^4 B}\int_0^{\bar{M}} dM \frac{M(4M^2+5X^2)}{(M^2+X^2)^{3/2}}\left[\frac{2}{3}M^3 - \frac{1}{2}M + \frac{1}{2\pi}\sum_{s=1} s^{-3/2} f_{\rm os}(s,M)\right] \\ &- \frac{v_b}{\pi^2\ell_{B'}^4 B}\frac{\bar{M}^2}{\sqrt{\bar{M}^2+X^2}}\left[\frac{2}{3}\bar{M}^3 - \frac{1}{2}\bar{M} + \frac{1}{2\pi}\sum_{s=1} s^{-3/2} f_{\rm os}(s,\bar{M})\right] \\ &- \frac{4\pi^2 v_b \ell_{B'}^2 N_c^2}{B}\frac{1}{\sqrt{\bar{M}^2+X^2}}\left\{\frac{2}{3}\bar{M}^3 - \frac{1}{2}\bar{M} + \frac{1}{2\pi}\sum_{s=1} s^{-3/2} f_{\rm os}(s,\bar{M})\right\}, \end{aligned} \tag{71}$$

with $X = m\ell_{B'}/(\sqrt{2}v_b)$

$$\bar{M} = \frac{\sqrt{2}\ell_{B'}}{2v_b}\sqrt{E_{\rm F}^2 - m^2}, \tag{72}$$

$$E_{\rm F} = \sqrt{4\pi^4 v_b^2 \ell_{B'}^4 N_c^2 + m^2}. \tag{73}$$

And

$$M_{c,T} = \frac{1}{\Delta}\frac{\partial\Delta}{\partial\theta}M_{c,\parallel}. \tag{74}$$

For the valence bands in the case when $m \ll \Lambda$

$$\begin{aligned} M_{v,\parallel} =& -\frac{\Lambda_m^2}{4v_b\pi^2\ell_{B'}^2 B} - \frac{v_b}{12\pi^2\ell_{B'}^4 B}\left(\ln\frac{\Lambda+\sqrt{\Lambda^2-m^2}}{\sqrt{2}v_b/\ell_{B'}} + \mathcal{A} - \frac{1}{4}\right) \\ &+ \frac{v_b}{8\pi^3\ell_{B'}^4 B}\sum_{s=1}^{\infty}\frac{1}{s^2}\sin\left(s\frac{\pi m^2\ell_{B'}^2}{v_b^2}\right) - \frac{m^2}{16\pi^2 v_b \ell_{B'}^2 B}\sum_{s=1}^{\infty}\frac{1}{s}\cos\left(s\frac{\pi m^2\ell_{B'}^2}{v_b^2}\right) \\ &- \frac{4\pi^4 v_b^2\ell_{B'}^4 N_c^2}{E_{\rm F} B}N_v. \end{aligned} \tag{75}$$

However, for a large gap m, the valence contribution is given by

$$\begin{aligned} M_{v,\parallel} =& \frac{v_b}{4\pi^3\ell_{B'}^4 B}\sum_{s=1}^{\infty}\frac{1}{s}\int_0^{\bar{n}} dn \sin(2\pi sn)\left[\frac{(2nv_b^2/\ell_{B'}^2)\Lambda}{(2nv_b^2/\ell_{B'}^2+m^2)\sqrt{\Lambda^2-m^2-2nv_b^2/\ell_{B'}^2}}\right. \\ &\left. - 4\ln\frac{\Lambda+\sqrt{\Lambda^2-m^2-2nv_b^2/\ell_{B'}^2}}{\sqrt{2nv_b^2/\ell_{B'}^2+m^2}}\right]. \end{aligned} \tag{76}$$

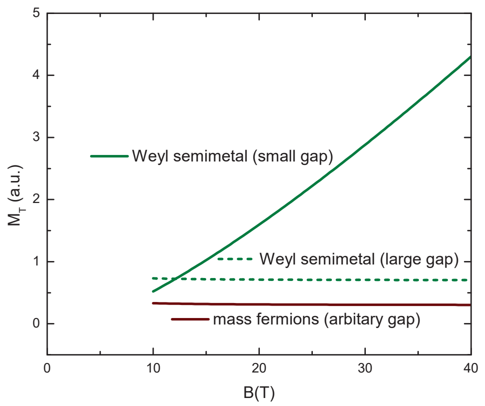

Supplementary Fig. 6. The schematic of the effective transverse magnetization M_T for gapped trivial electron gases and Weyl semimetals in the quantum limit for a fixed electron density N_c.

If the gap is so large that $\sqrt{\Lambda^2 - 2v_b^2/\ell_{B'}^2} < m < \Lambda$, the above expression reduces to

$$M_{v,\parallel} = \frac{e\Delta}{16\pi^2 v_b \hbar}\left(m^2 \ln \frac{\Lambda + \sqrt{\Lambda^2 - m^2}}{\Lambda - \sqrt{\Lambda^2 - m^2}} - 2\Lambda\sqrt{\Lambda^2 - m^2}\right) = \text{constant}. \tag{77}$$

Further, for the effective transverse magnetization

$$M_{v,T} = \frac{1}{\Delta}\frac{\partial \Delta}{\partial \theta} M_{v,\parallel}. \tag{78}$$

Therefore the total magnetization shown in Supplementary Fig. 6 still does not saturate for small gaps but tends to saturate at very large gaps, following the relations

$$M_\parallel \propto \begin{cases} -B\ln\sqrt{B}, & m \ll \Lambda, \\ \text{constant}, & \sqrt{\Lambda^2 - 2v_b^2/\ell_{B'}^2} < m < \Lambda, \end{cases} \tag{79}$$

$$M_T \propto \begin{cases} B\ln\sqrt{B}, & m \ll \Lambda, \\ \text{constant}, & \sqrt{\Lambda^2 - 2v_b^2/\ell_{B'}^2} < m < \Lambda. \end{cases} \tag{80}$$

Field-dependent magnetization of TaAs based on its real band structure. For the case of two branches of Weyl fermions with the Weyl nodes denoted as W1 and W2, respectively, the total thermodynamic potential $\Omega(B)$ can be written as the summation of the contributions of W1 and W2, i.e.,

$$\Omega(B) = \Omega_1(B) + \Omega_2(B). \tag{81}$$

Therefore, the magnetization is given by

$$M_{\parallel} = -\frac{\partial \Omega}{\partial B} = -\frac{\partial \Omega_1}{\partial B} - \frac{\partial \Omega_2}{\partial B} = M_{\parallel}^{(1)} + M_{\parallel}^{(2)}, \tag{82}$$

$$M_T = -\frac{1}{B}\frac{\partial \Omega}{\partial \theta} = -\frac{1}{B}\frac{\partial \Omega_1}{\partial \theta} - \frac{1}{B}\frac{\partial \Omega_2}{\partial \theta} = M_T^{(1)} + M_T^{(2)}. \tag{83}$$

For the present TaAs, the carriers of W1 and W2 are electrons. Hence, for a fixed electron density in the quantum limit we have

$$M_{\parallel} \propto -\sum_{i=1}^{2} B \ln\left(\frac{2\Lambda_i \ell_B}{v_{bi}\sqrt{2\Delta_i}}\right), \tag{84}$$

$$M_T \propto -\sum_{i=1}^{2} B \ln\left(\frac{2\Lambda_i \ell_B}{v_{bi}\sqrt{2\Delta_i}}\right)\frac{\partial \Delta_i(\theta)}{\partial \theta}. \tag{85}$$

Here

$$\Delta = \frac{v_{ai}}{v_{bi}}\cos\alpha_i \cos\theta + \frac{v_{ci}}{v_{bi}}\sin\alpha_i \sin\theta, \tag{86}$$

$$\alpha_i = \tan^{-1}\left(\frac{v_{ci}}{v_{ai}}\tan\theta\right), \tag{87}$$

and $-\Lambda_i$ is the minimum energy for the ith Weyl fermions.

Supplementary Note 3: Outline of Fermi surface mapping and band calculations

Supplementary Fig. 7 shows the main results of the analyses on the dHvA oscillations of TaAs. In order to obtain the oscillatory component, the non-oscillatory background was subtracted by polynomial fittings. The subtracted dHvA oscillations at different angles are shown in Supplementary Fig. 7a, where the data are shifted randomly for a clear presentation. The normal fast Fourier transform (FFT) shows the changes of frequency peaks in different angles. There is indeed only one base frequency (F_α) at angles smaller than 34.5°, which is 8.1 T at angle θ ∼12°. So the QL of TaAs is small enough for approaching in a constant high magnetic field in lab. There emerges another small frequency F_β at higher angles which shows comparably less angle dependence. The effective mass can be extracted from the amplitudes of the oscillatory component at different temperatures (Supplementary Fig. 7c). The FFT amplitudes are obtained from a field window 3 – 15 T and plotted against temperature as shown in Supplementary Fig. 7d. The effective mass is estimated

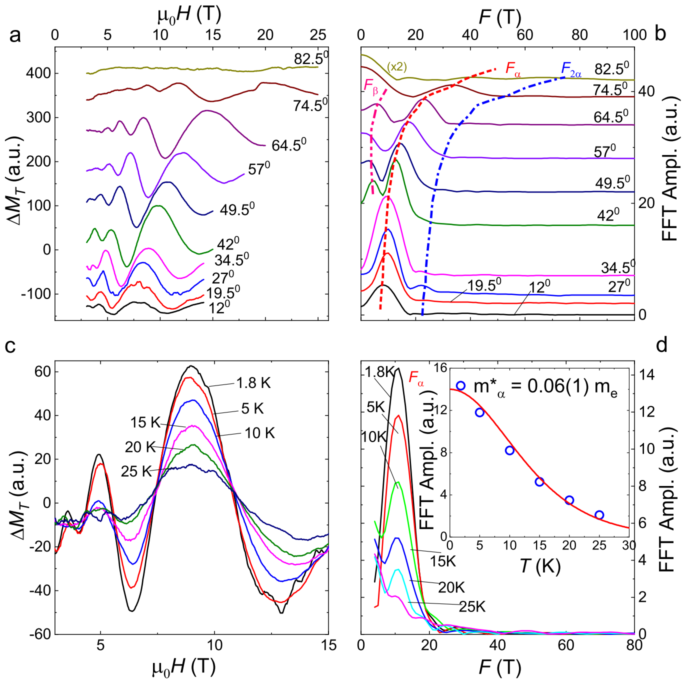

Supplementary Fig. 7. **Analyses of dHvA oscillations. a** dHvA oscillations at different angles. The curves are shifted for clearness. **b** FFT analysis at each angle. The red dashed and blue point-dashed lines track the base frequency F_α and its doublet $F_{2\alpha}$, respectively. The pink point-dashed line tracks the frequency F_β which originates from the pocket near W2. **c** dHvA oscillations at different temperatures for $\theta \sim 34.5°$. **d** The FFT amplitudes at different temperatures. The inset shows the fitting result of effective mass.

as 0.06 m_e by fitting the amplitude factor $A(B,T) \propto \exp(-2\pi^2 k_B T_D/\hbar\omega_c)\frac{\lambda(T)}{\sinh(\lambda(T))}$ of L-K formula, where T_D is the Dingle temperature, the cyclotron frequency $\omega_c = eB/m_{\rm cyc}$ and $\lambda(T) = 2\pi^2 k_B T/\hbar\omega_c$. We can also obtain the carrier density for the two Weyl pockets based on the angle-dependent dHvA data. By an ellipsoid approximation, the carrier density of the electron pocket contains the W1 is estimated as $n_{W1} = k_{Fa}^2 k_{Fc}/3\pi^2 = 3.96\times10^{17}$ cm^{-3} per pocket. The carrier density of the electron pocket contains the W2 is estimated as $n_{W2} = k_{Fa}^3/3\pi^2 = 3.82\times10^{16}$ cm^{-3} per pocket. All the results are consistent with those in Ref. [7].

The useful information obtained from the quantum oscillations give us a comprehensive view of the band structure of TaAs. We performed the first-principle calculations by the OPENMX code within the framework of the generalized gradient approximation of density-

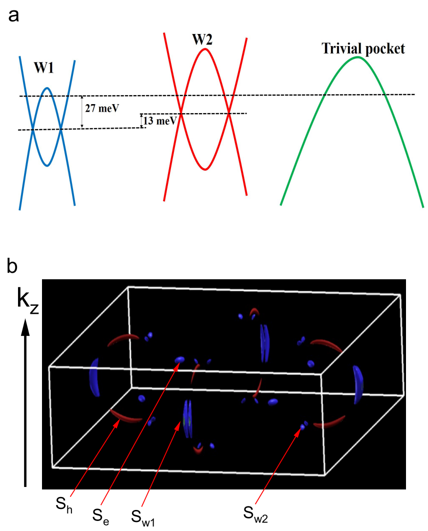

Supplementary Fig. 8. **Band structure for TaAs. a** A simple sketch of the band distribution in TaAs. The Fermi energy is placed 27 meV above W1 based on dHvA data. **b** The carrier pockets in a conventional cell.

functional theory(DFT) [8]. Experimental lattice parameters were used from Ref. [9], where the details of the calculations can be found in our previous work [10].

Supplementary Fig. 8a shows a brief sketch of band distribution in TaAs. The FL is placed 27 meV above W1 and the resulting carrier pockets in a conventional cell look like that in Supplementary Fig. 8b. The **x-y** plane cross-sectional area is calculated as shown in Supplementary Table. 1, and these results fit well with the two main frequencies resolved in the dHvA data.

Supplementary Table 1. Electron (e) and hole (h) maximal areas (S) of cross sections on **x-y** plane. $S_{W1}(S_{W2})$ is the Fermi surface enclosing and evolved from the $W_1(W_2)$ Weyl point(s), and $S_e(S_h)$ is an electron (hole) pocket Fermi surface not evolved from any Weyl point. The units of area is 10^{-4}Å^{-2}.

	S_{W1}	S_{W2}	S_e	S_h
S_{xy}	8.9	4.1	18	10

Supplementary Note 4: The universality of the high-field criterion

In order to present a consistent picture, we also carried out the torque measurements on other compounds of the TaAs family. As shown in Supplementary Figs. 9a and 9b, the low-field torque of NbAs and TaP can be fitted by the conventional parabolic relation, but show different signals dependent on the certain compound. However, we can see the signals on the high-field side are the same and universal, and all are non-saturating in M_T plots (Supplementary Figs. 9c and 9d).

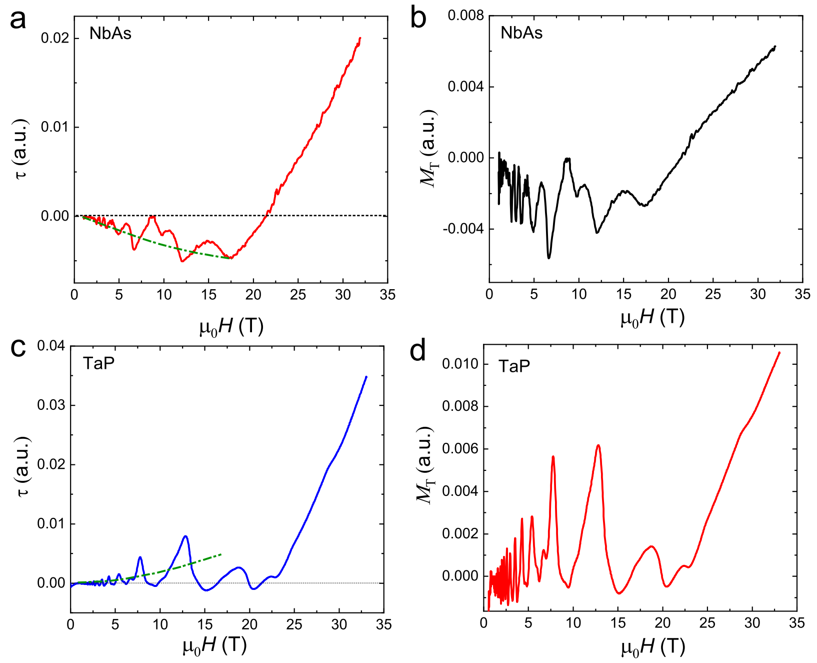

Supplementary Fig. 9. **The high-field torque of other members of the TaAs family.** Torques and corresponding transverse magnetizations (M_T) for NbAs **a** & **c** and TaP **b** & **d**, respectively. The low-field (below the QL) green lines represent the fitted parabolic non-oscillating backgrounds, which show different signals compared with the high-field data (in the high-field limit the data should always be diamagnetic) in different TaAs family members. However, we can see the signals on the high-field side are the same and universal, and all are non-saturating in the M_T plots.

Supplementary Note 5: SQUID measurement and the absolute value re-scaling

In order to obtain the absolute units of high-field parallel magnetization and further support the proof of the Landau-diamagnetism dominating physics (over the small core-shell contribution), we here show the low-field magnetic property measurement results carried out in SQUID. As shown in Supplementary Figs. 10a and 10c, the temperature-dependent magnetic susceptibility of TaAs for H $\parallel$ $\boldsymbol{c}$ and H $\perp$ $\boldsymbol{c}$ show similar profiles of diamagnetic signals and crystal orientation-dependent anisotropy. As shown in Supplementary Figs. 10b and 10d, magnetic-dependent magnetization of TaAs for H $\parallel$ $\boldsymbol{c}$ and H $\perp$ $\boldsymbol{c}$ show large diamagnetic signals and clear dHvA oscillations superposing on the linear background. All the low-field characterizations are consistent with the data in Ref.[11]. This detailed magnetic property characterization on our measured samples further show the strong evidence of the dominant role of Landau diamagnetism in TaAs. As shown in Supplementary Fig. 11, the absolute units for the pulsed data (high-field data) can be obtained by re-scaling the data against SQUID taken at low fields. Samples used in each measurement are from the same batch grown in one month, which makes the results consistent and fit well.

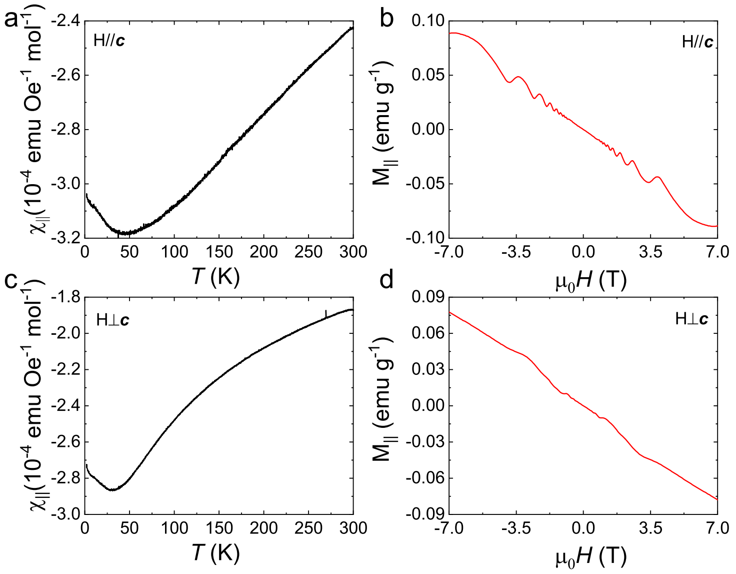

Supplementary Fig. 10. **Low-field magnetic property characterization (SQUID) of TaAs.** The temperature-dependent magnetic susceptibility of TaAs for H $\parallel$ $\boldsymbol{c}$ (**a**) and H $\perp$ $\boldsymbol{c}$ (**c**), respectively. The magnetic-dependent magnetization of TaAs for H $\parallel$ $\boldsymbol{c}$ (**b**) and H $\perp$ $\boldsymbol{c}$ (**d**), respectively.

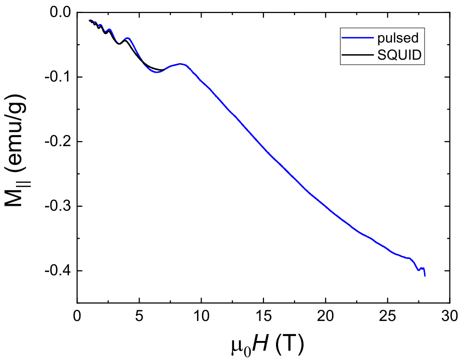

Supplementary Fig. 11. The correction of the high-field data by using the low-field SQUID measurements.

Supplementary Note 6: Details on the fitting for the high-field transverse and parallel magnetization

We can approximate $f_{\rm os}(1, M)$ as

$$f_{\rm os}(1, M) \simeq \frac{\sqrt{2}}{2} \sin\left(2\pi M^2 - \frac{\pi}{4}\right). \tag{88}$$

Hence, the conduction contributions are

$$M_{c,\parallel} \simeq -\frac{\sqrt{\Delta}\Delta E_{\rm F}}{8\pi^3 \ell_B^3 B} \sin\left(2\pi \frac{E_{\rm F}^2 \ell_B^2}{2\Delta v_b^2} - \frac{\pi}{4}\right), \tag{89}$$

$$M_{c,T} \simeq \left[-\frac{E_{\rm F}^2}{8\pi^2 v_b \ell_B^2 B} - \frac{\sqrt{\Delta} E_{\rm F}}{8\pi^3 \ell_B^3 B} \sin\left(2\pi \frac{E_{\rm F}^2 \ell_B^2}{2\Delta v_b^2} - \frac{\pi}{4}\right)\right] \frac{\partial \Delta(\theta)}{\partial \theta}. \tag{90}$$

We introduce an exponential decay of the oscillation parts due to the impurity scattering and electron-electron interaction, similar to the Dingle factor $\exp(-\Gamma/B)$ [4] with Γ being a constant. By adding the log-form of the valence contribution, we obtain two equations to fit the experimental data

$$M_{\parallel} \simeq -\frac{\sqrt{\Delta}\Delta E_{\rm F}}{8\pi^3 \ell_B^3 B} \sin\left(2\pi \frac{E_{\rm F}^2 \ell_B^2}{2\Delta v_b^2} - \frac{\pi}{4}\right) \exp\left(-\frac{\Gamma}{B}\right) - \frac{v_b \Delta^2}{24\pi^2 \ell_B^4 B}\left[\ln\left(\frac{2\Lambda}{v_b\sqrt{2\Delta e B}}\right) + \mathcal{A} - \frac{1}{4}\right], \tag{91}$$

$$M_T \simeq \left[-\frac{E_{\rm F}^2}{8\pi^2 v_b \ell_B^2 B} - \frac{\sqrt{\Delta} E_{\rm F}}{8\pi^3 \ell_B^3 B} \sin\left(2\pi \frac{E_{\rm F}^2 \ell_B^2}{2\Delta v_b^2} - \frac{\pi}{4}\right) \exp\left(-\frac{\Gamma}{B}\right)\right] \frac{\partial \Delta(\theta)}{\partial \theta} - \frac{v_b \Delta}{24\pi^2 \ell_B^4 B}\left[\ln\left(\frac{2\Lambda}{v_b\sqrt{2\Delta e B}}\right) + \mathcal{A} - \frac{1}{4}\right] \frac{\partial \Delta(\theta)}{\partial \theta}. \tag{92}$$

[1] C. Zhang, *et al.*, Signatures of the Adler-Bell-Jackiw chiral anomaly in a Weyl fermion semimetal, Nature Commun. **7**, 10735 (2016).

[2] L. Li, J. Checkelsky, Y. Hor, C. Uher, A. Hebard, R. Cava, and N. Ong, Phase transitions of dirac electrons in bismuth, Science **321**, 547 (2008).

[3] L. Li, *Torque magnetometry in unconventional superconductors* (Princeton University, 2008).

[4] D. Shoenberg, *Magnetic oscillations in metals* (Cambridge University Press, 2009).

[5] N. F. Mott and H. Jones, *The theory of the properties of metals and alloys* (Courier Corporation, 1958).

[6] G. P. Mikitik and Y. V. Sharlai, Magnetic susceptibility of topological nodal semimetals, Phys. Rev. B **94**, 195123 (2016).

[7] F. Arnold, M. Naumann, S.-C. Wu, Y. Sun, M. Schmidt, H. Borrmann, C. Felser, B. Yan, and E. Hassinger, Chiral Weyl pockets and Fermi surface topology of the Weyl semimetal TaAs, Phys. Rev. Lett. **117**, 146401 (2016).

[8] J. P. Perdew, K. Burke, and M. Ernzerhof, Generalized gradient approximation made simple, Phys. Rev. Lett. **77**, 3865 (1996).

[9] J. Murray, J. Taylor, L. Calvert, Y. Wang, E. Gabe, and J. Despault, Phase relationships and thermodynamics of refractory metal pnictides: The metal-rich tantalum arsenides, Journal of the Less Common Metals **46**, 311 (1976).

[10] S.-M. Huang, *et al.*, A Weyl fermion semimetal with surface Fermi arcs in the transition metal monopnictide TaAs class, Nature Commun. **6**, 7373 (2015).

[11] Y. Liu, Z. Li, L. Guo, X. Chen, Y. Yuan, F. Liu, S. Prucnal, M. Helm, and S. Zhou, Intrinsic diamagnetism in the Weyl semimetal TaAs, J. Magn. Magn. Mater. **408**, 73 (2016).
